# Strong positively diversity–productivity relationships in the natural sub-alpine meadow communities across time are up to superior performers

**DOI:** 10.1038/s41598-020-70402-6

**Published:** 2020-08-07

**Authors:** Kai Jiang, Zhaoyuan Tan, Qifang He, Lu Wang, Yang Zhao, Xinhang Sun, Weichen Hou, Wenxing Long, Hui Zhang

**Affiliations:** 1 College of Forestry/Wuzhishan National Long Term Forest Ecosystem Monitoring Research Station, Hainan University, Haikou, 570228 People’s Republic of China; 2 Key Laboratory of Genetics and Germplasm Innovation of Tropical Special Forest Trees and Ornamental Plants (Hainan University), Ministry of Education, College of Forestry, Hainan University, Haikou, 570228 People’s Republic of China

**Keywords:** Biodiversity, Community ecology, Ecosystem ecology

## Abstract

In experiments that test plant diversity–productivity relationships, the common practice of weeding unsown species and disallowing species colonization may have the unintended consequence of favoring priority effects that maintain niche complementarity in determining productivity. However, in naturally assembled communities where colonization occurs, resource competition may favor dominant traits, which eventually have the greatest influence on productivity. Here, in naturally developed long-term subalpine meadows (from 4-year to at least 40 years meadows) in the Qinghai-Tibetan Plateau, we investigated the relationships between species richness and productivity to testify whether positive diversity–productivity relationships can still exist in naturally developed long-term communities. We also measured five functional traits (specific leaf area, photosynthesis rate, leaf proline content, seed mass and seed germination rate) to calculate two functional diversity indices: community-weighted mean trait values (CWM) and Rao’s quadratic entropy (RaoQ) which are highly correlated to functional traits of dominating species and variety of functional trait among all species. Finally, we quantified the relative contribution of species diversity, functional traits of dominating species and functional diversity among all species to productivity along the succession. We demonstrated strong positively diversity–productivity relationships in the natural sub-alpine meadow communities across time. The five traits of dominating species explained a large proportion (54–80%) of the variation in productivity during succession, whereas species diversity and functional diversity (FD) for each of the five traits explained much less (24–48% for species richness and 0–40% for FD for each of the five traits respectively). We found unequivocal evidence that significantly positive diversity–productivity relationships in the natural sub-alpine meadow communities across time are up to superior performers (dominant traits) in naturally developed communities where colonization occurs. As a result, understanding diversity–productivity relationships under the full range of community assembly processes therefore merits further investigation.

## Introduction

Global biodiversity is declining sharply^1^ with the potential to impair ecosystem functioning in the near future, but the mechanisms that connect biodiversity to ecosystem function are not well understood^2^. In diversity–function relationships, the connection between plant diversity and productivity is considered particularly important^3^. Although the classic diversity–productivity relationship is thought to be hump-shaped, with species richness highest at intermediate levels of productivity^4^, experimental studies have, however, mostly yielded linear positive diversity–productivity relationship^5–9^. In long-term experiments, the positive diversity–productivity relationships have even been observed to become stronger over time^10,11^, reinforcing the idea that multispecies communities tend to perform better than the average monoculture.

The contribution to this so-called ‘Net Biodiversity Effect’ may come through a combination of niche complementarity that allows coexisting species to better exploit available resources, or simply through the dominant effects of superior performers or high-fitness species (i.e., the selection effect). Empirical evidence overwhelmingly points to the role of niche complementarity, particularly in high diversity communities, but multispecies assemblages were rarely more productive than the monoculture of the most productive species^12^. Given enough time however, as long-term experiments show, multispecies assemblages tended to outperform monocultures of even the most productive species, indicating that the magnitude of the complementarity effect increased with time^13^. Despite this emerging clarity on diversity–productivity relationships (DPRs), the difficulty in predicting the combinations of species that contribute to maximizing ecosystem productivity through the complementarity effect is not straightforward. It has been argued that functional diversity take these life history and trait differences into account, such as the community weighted mean (CWM), which captures the traits of the dominant species, or the diversity of trait values among species (functional trait diversity, FD) in the community, could better capture the contributions of the different species to productivity. The effects of CWM and FD for traits on productivity can be reflected by biomass ratio hypothesis (productivity are determined by the presence or absence of highly productive species) and niche complementarity hypothesis (productivity are determined by the variety and complementarity of species) respectively^13,14^. In fact, niche complementarity invokes functional dissimilarity (FD), and it is easy to appreciate that such FDs can exploit environmental heterogeneity and contribute to greater ecosystem functioning than equal numbers of functionally redundant species^13^.

However, extrapolating the patterns of diversity–productivity relationships found in experimentally constructed communities to natural communities might be difficult^15,16^. That is because a common feature of experimental investigations on DPRs is that they involve the weeding out of non-study species that colonize the study plots. Consequently, any successional change within the experimental plots is not allowed in such experiments^17^. As a result, processes such as dispersal, colonization, environmental filtering, and competition may not be represented in experimental communities in the same way they contribute to the structure of natural long-term communities^18^.

Furthermore, the timescale is also important for affecting diversity–productivity relationships, as the evidence from some non-weeded biodiversity–functioning experiments show that diversity–biomass relationships weaken over time^17^ due to species colonization and turnover and the progressive increase in the proportions of the few productive species. It therefore remains unclear whether a positive diversity–biomass relationship is the norm in communities that are subject to colonization events and species replacement over time.

Secondary succession may provide a useful opportunity to test diversity–productivity relationships in naturally assembled communities for the following three reasons. First, succession usually results in an increase in species richness over time^19^, which provides a natural gradient in species richness to test its influence on productivity^20–23^. Second, the abiotic environment (e.g., soil nutrients and light) also varies over succession, influencing functional and species diversity^24,25^. Thus, the relationships between species richness, FD, CWM, and productivity as a function of variation in the abiotic environment can also be quantified. Third, while we can understand how biodiversity loss decreases ecosystem functioning at the local scales at which species interact, it remains unclear how biodiversity loss affects productivity at the larger spatial and temporal scales^26^. Testing positive diversity–productivity relationships and their underlying mechanisms in secondary succession can help unravel how positive diversity–productivity relationships change with time^13,27^.

Here, we studied plant diversity -aboveground biomass relationships in sub-alpine meadow plant communities in the Qinghai-Tibetan Plateau. Our study site is a well-researched secondary successional chronosequence, with meadows that include sites undisturbed for at least 40 years, to those that were farmed but have now been protected from agricultural exploitation for 4-, 6-, 10-, and 13-years^19,23^. Using this system we attempt to answer the following questions: i) whether a consistent positive relationships between species richness and productivity can be found in successional communities; and ii) the relative contributions of functional traits of dominant species, species richness and niche complementarity to productivity in naturally developed communities over time.

## Materials and methods

### Study site

Our study site is the species-rich sub-alpine meadows located in the eastern part of the Qinghai-Tibetan plateau, Hezuo, China (34°55**′**N, 102°53**′**E) with mean elevation approximately 3000 m above sea level. Although the Tibetan Plateau Monsoon and Asian Monsoon^28^ brings rain, the study region has cold and dry climate, with mean annual temperature of 2.4° C and mean annual precipitation of just 530 mm^23^. The vegetation is dominated by herbaceous species such as *Elymus nutans* Griseb (Poaceae), *Kobresia humilis* (C.A. Mey.) Serg*.* (Cyperaceae) and *Thermopsis lanceolata* R. Br. (Fabaceae)^23^. Human impacts include agricultural exploitation and pastoralism are the primary current land use, which in places have caused serious land degradation. In response, local governments have stopped further agricultural exploitation and constructed fences to restrict livestock grazing. These efforts gave rise to successional chronosequences, such as the ones we use in our study.

**Figure 1:**
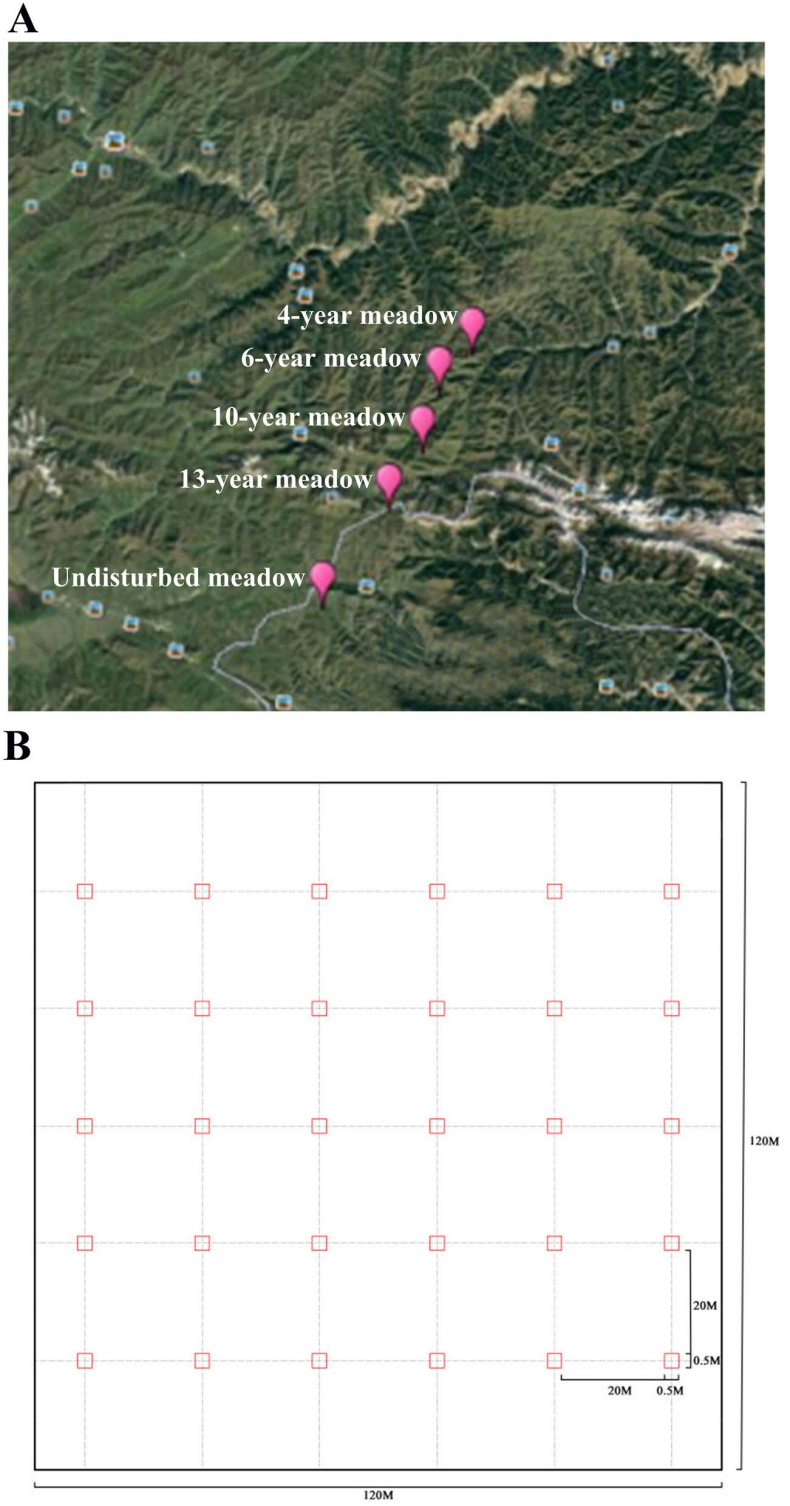
Location map of our study sites and our quadrat sampling design. (**A**) locations of five sites representing each of the five successional ages (4-, 6-, 10-, 13-year and undisturbed grassland), (**B**) the 30 0.5 × 0.5 m^2^ quadrats sampling design in each of the five successional meadows. The map of Fig. 1A was obtained from Google Earth online version (https://earth.google.com/, access on 12/10/2018). Figure labels on the map were added using Google Earth online toolkit and text labels using Windows image processing software Paint.

We identified a chronosequence of fields that had been undisturbed for 4-, 6-, 10-, 13-, and 40-years (the control)^19,23^. All our sample sites, except for the control meadows, had been used for agriculture to grow highland barley in the recent past, with cessation of cultivation within the last 4–13 years. The time since last agricultural use was determined by interviews with local farmers. There are 1–10 km apart among the five meadows and all meadows possessed comparable topographic characteristics (e.g., orientation and slope), soil types and climate (Fig. 1A). This chronosequence is one of the same chronosequence in our previous work^23^ and we have observed that species richness increased from 61 to 82 species during succession, with 50 species sharing among all five successional meadows. Species composition was similar between 4-year and 6-year meadows, with 60 species sharing between these two meadows. Similar patterns were found in late successional meadows, with 70 species shared among 10-year, 13-year and undisturbed meadows.

### Field sampling

The vegetation in each field was sampled in August 2013. An area of 120 × 120 m^2^ was randomly selected in each meadow. Within this area, thirty 0.5 × 0.5 m^2^ quadrats were regularly arranged in six parallel transects, with 20 m intervals between each two adjacent quadrats (detail please see Fig. 1B). To determine species richness and abundances, in each quadrat we recorded all the aboveground ramets and identified them to species.

To determine aboveground biomass, we removed all the ramets in each quadrat and took them to the laboratory, where they were oven-dried at 100℃ for 2 days and then weighed. Productivity is typically the amount of carbon fixed per unit time, not standing biomass. Here we follow methods of previous diversity–productivity studies in grasslands^29,30^, which have used aboveground biomass as proxy for productivity.

### Functional trait data collection

We quantified the carbon economy of leaves by measuring specific leaf area (SLA, cm^2^ g^−1^). We quantified light capture strategy via photosynthesis rate (A, u mol^−1^). We estimated resistance to abiotic stress via leaf proline content (Pro, mg/kg), seed mass (SM, g) and seed germination rate (SG, %). Importantly, the functional traits for the same species at each successional age separately if they occurred in multiple meadows were measured to ensure that successional age-related intraspecific variation was appropriately incorporated into our analyses. All functional traits were determined as described in our previous work^19,22,23^ and the detailed procedures were given in the Supplementary Material.

### Statistical methods

First, we compared variation during successional change in the proportion of total biomass for the three main functional groups of plants: forbs (dominant in early succession), legumes, and graminoids (both dominant in later succession) to check whether there are significant turnovers in the dominant plant taxa from early to late succession. Then, we used Spearman correlation analysis to quantify whether significantly positive correlations between empirical species diversity (S, numbers of species richness per square meters) and productivity (aboveground biomass per square meters, P) can be observed in each successional meadow.

For each of the five functional traits (SLA, A, Pro, Sm, and SG), we calculated two functional diversity indices: the community-weighted mean (CWM) and functional diversity (FD) represented by Rao’s quadratic entropy (RaoQ).

The two indices were calculated as follows:

1 $$ {CWM} = \sum\limits_{i = 1}^{n} {p_{ij} \times t_{ij} } $$

where *p*_*ij*_ is the relative abundance of the species *i* in each 0.5 × 0.5 m^2^ quadrat *j*, and *t*_*ij*_is the mean trait value of the species *i* in each successional meadow *j*.

2 $$ RaoQ_{i} = \sum\limits_{i = 1}^{n} {\sum\limits_{i = 1}^{n} {p_{i} \times p_{k} \times d_{ik} } } $$

where *p*_*i*_ and *p*_k_are the relative abundance of species *i* and *k* in each 0.5 × 0.5m^2^ quadrat *j* respectively and *d*_*ik*_ is the dissimilarity coefficient based on Euclidean distance between two species *i* and *k* in the multivariate trait space of each successional meadow *j*.

Then, a variance partitioning analysis was used to test the relative contributions of species richness, the CWM and FD represented by RaoQ of these five traits to productivity in each successional meadow. We also used variance partitioning to allocate changes in productivity in each successional meadow arising from four complementary components: (a) variation explained by species richness, (b) variation explained CWM of each of the five traits, (c) variation explained by FD of each of the five traits only, and (d) “unexplained variation”^31^. Across all successional meadows, species richness, and aboveground biomass, CWM and FD of all five traits (SLA, A, Pro, SM, and SG) were strongly right-skewed, so we log-transformed species richness, and aboveground biomass, CWM and FD of all five traits to meet the assumption of normality required by variance partitioning. At each successional meadow, variance partitioning was done using the function of “varpart” in “vegan” package in R^32^. All analyses above were performed in R (R Core Team 2019).

## Results

**Figure 2:**
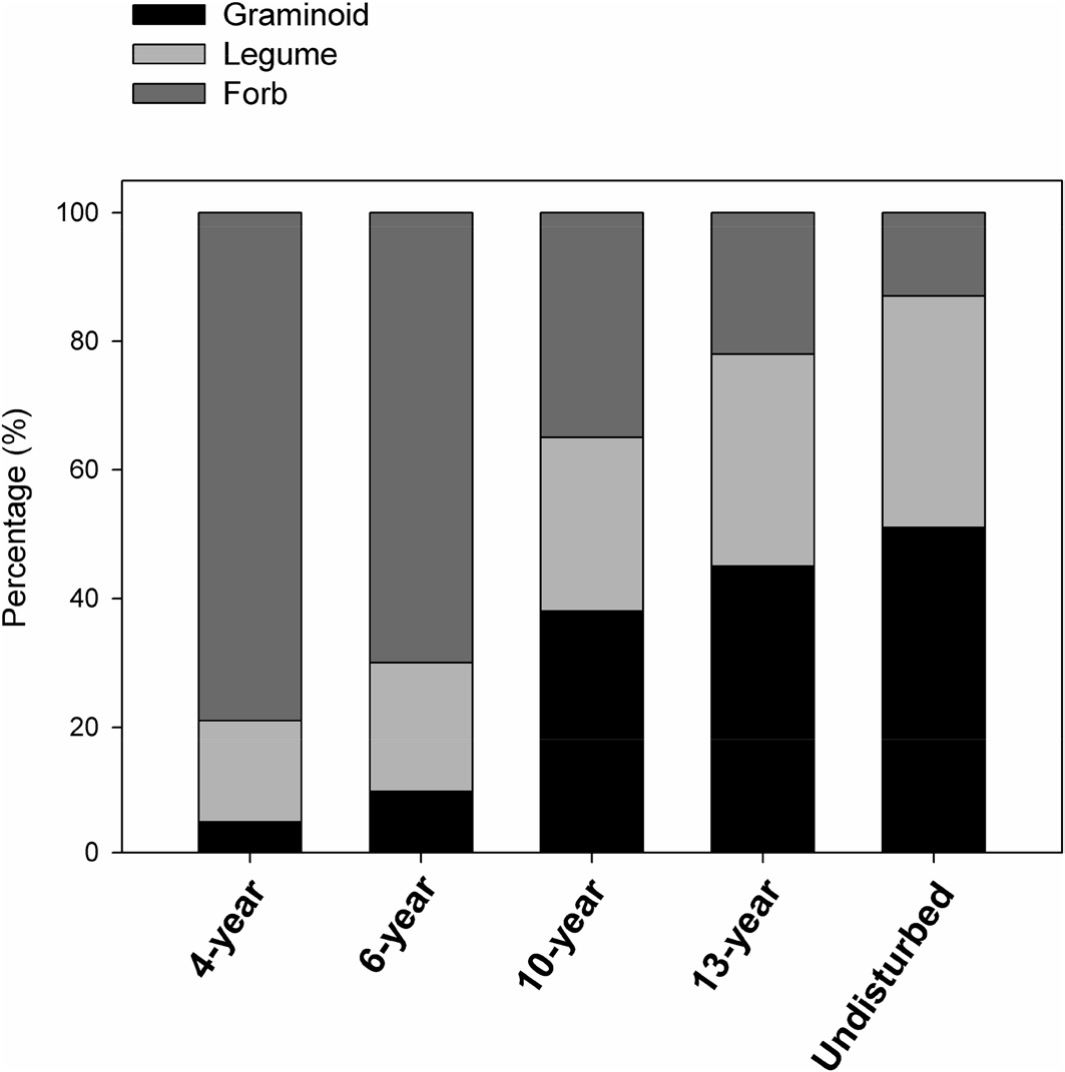
The respective percentages of the total biomass for the three main function group (forb, graminoid and legume species) among the 30 quadrats in each successional age.

Forbs accounted for the greatest relative biomass in early succession fields (4- and 6-year meadow), but decreased from 79 to 13% in our successional chronosequence (Fig. 2). In contrast, grasses accounted for most biomass in late succession (10-, 13- year and undisturbed meadows), increasing from 5% in early succession to 51% in late succession (Fig. 2). During the same period, legume species increased in relative abundance from 16 to 36% (Fig. 2). Thus, there are significant turnovers in the dominant plant taxa from early to late succession.

**Figure 3:**
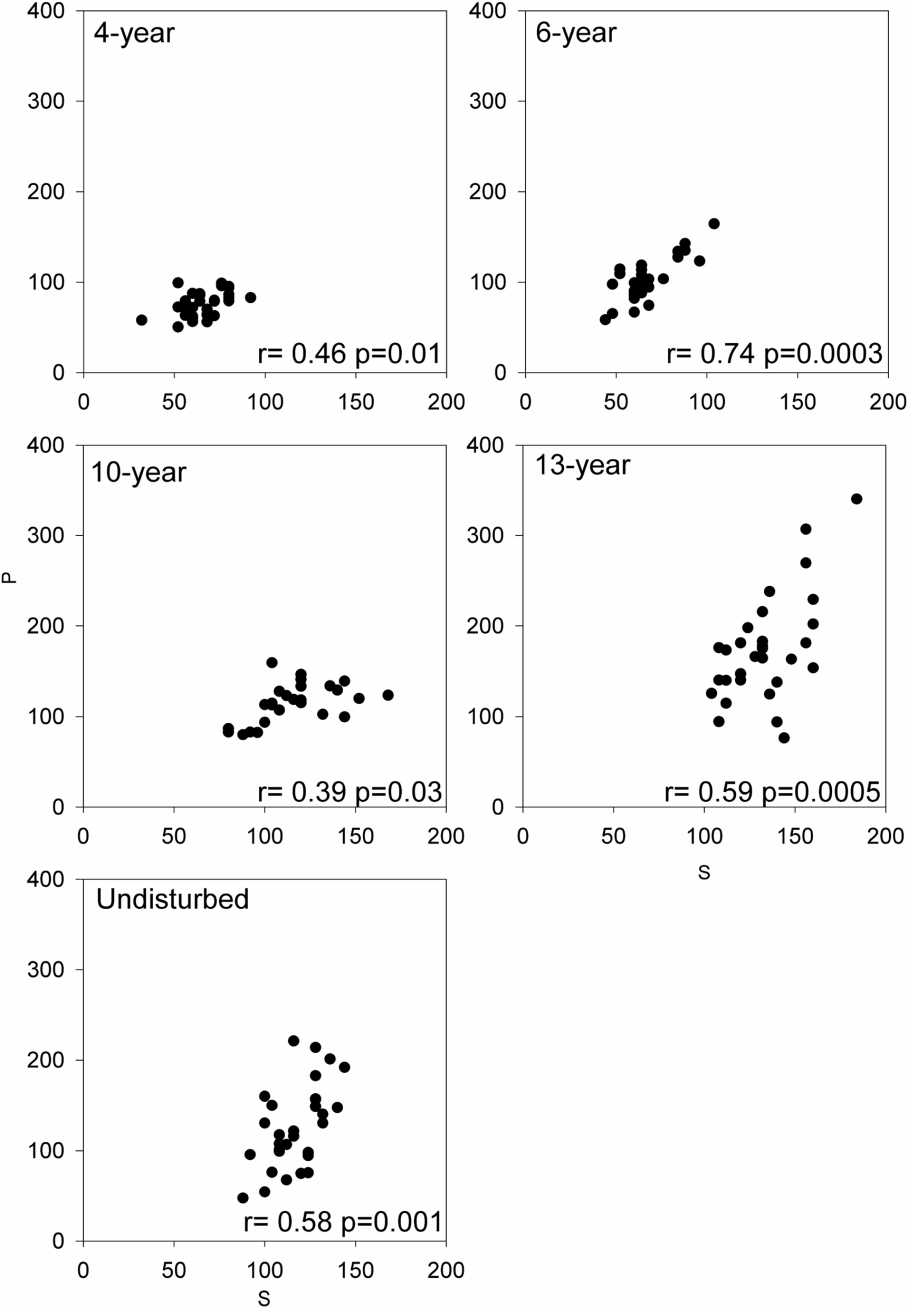
The relationships between empirical species diversity (S; numbers of species per square meters) and productivity (P, g per square meters)) along the successional gradient. Each point represents S and P in each of 30 0.25 m^2^ quadrats in each successional meadow.

Both early-successional (4- and 6-year meadow) and late-successional (10-, 13-year, and undisturbed) meadows showed consistently and significantly positive relationships between productivity and species richness (Fig. 3).

**Figure 4:**
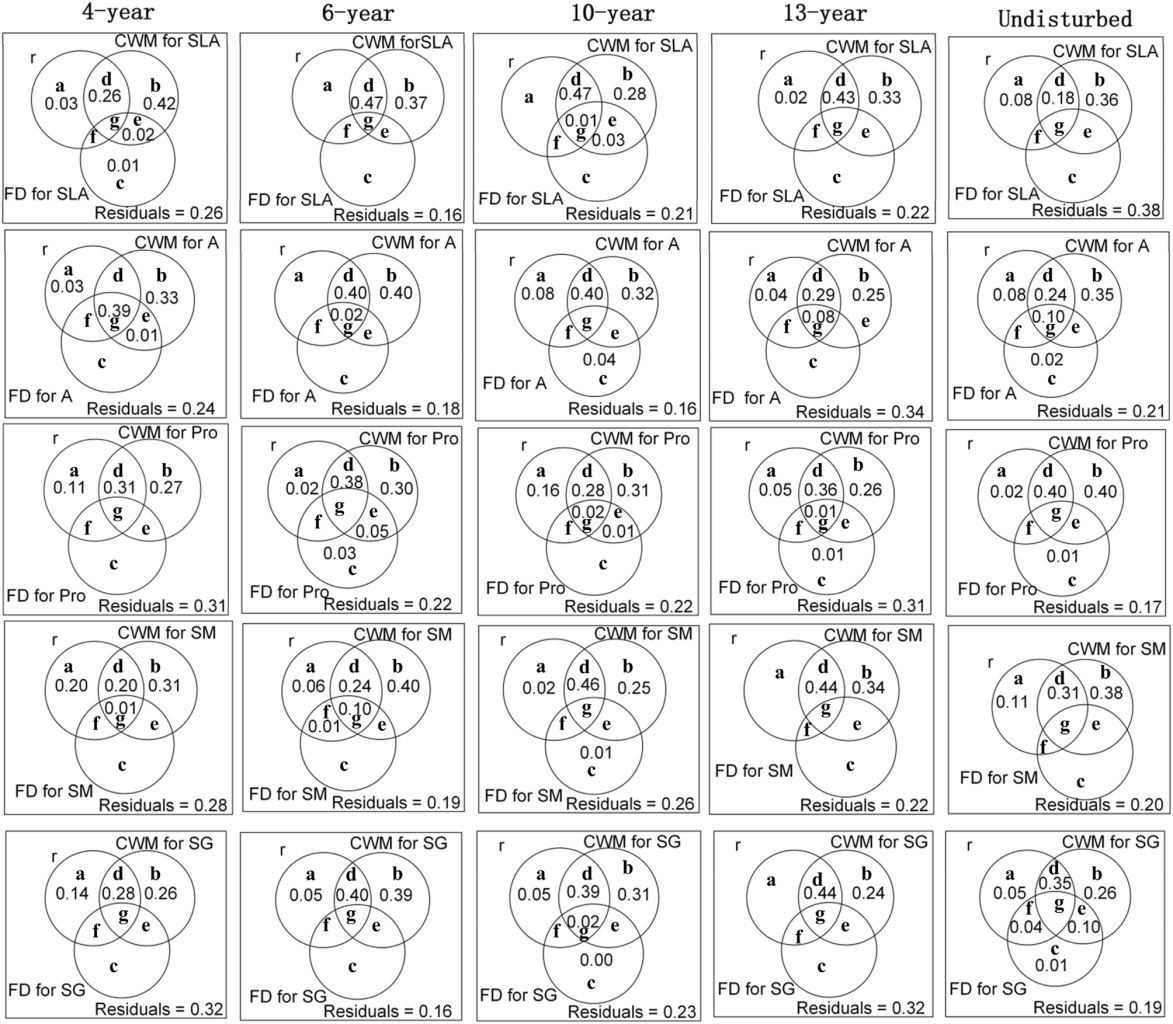
Variation in aboveground biomass along a successional gradient, partitioned into species richness (r), CWM for each of the five functional traits, FD for each of the five traits, and undetermined variation (Residuals). Traits are: specific leaf area (SLA), leaf photosynthesis rate (A), leaf proline content (Pro), seed mass (SM) and seed germination rate (SG). a + d + f + g represents variations of productivity explained by species richness (r). d + b + g + e indicates variations of productivity explained by CWM for each of the five traits. f + g + e + c showes variations in productivity explained by FD for each of the five traits.

Our variance partitioning analysis shows that CWMs for each of the five traits explained a large proportion (54–80%) of the variation in productivity during succession, whereas species richness and FDs for each of the five traits explained much less (24–48% for species richness and 0–40% for FDs for each of the five traits respectively) (Fig. 4). As a result, CWMs for each of the five traits Determine productivity over succession.

## Discussion

We found consistent positive relationships between plant diversity and productivity in successional grasslands with a wide range of successional community ages (4 to > 40 years) in a sub-alpine meadow ecosystem. Our results reinforce arguments^13^ for positive relationships between plant diversity and productivity, and also expand the findings from artificial experiments in relative short-term grasslands to naturally assembled long-term meadow plant communities.

The relationship between species richness and productivity is of enormous significance to both fundamental and applied aspects of ecology, and has been a persistent subject of interest and debate^5,29,33^. A number of previous studies have shown that greater plant diversity can promote greater productivity^9,10,17,34^. However, these results were derived mainly from controlled experiments in the relatively short-term (usually less than 10 years), and data from long-term experiments (greater than 10 years) or from natural ecosystems where the full range of ecological processes (e.g., colonization) occur are rare^27^. Could the observed positive relationship between species richness and productivity from short-term experiments therefore merely reflect transient dynamics in limited experimental designs^18^ and not the patterns that are to be found in natural old-growth communities^15,16^? Or do they reflect more general processes that are widely applicable? Our results provide a consistent and robust picture of this important relationship in a natural ecosystem, and expand the scope of positive diversity–productivity relationships to a wider range of communities.

Our variance partitioning results showed that CWMs for the five traits (SLA, leaf proline content, photosynthesis rate, seed mass and seed germination rates) showed much higher predictive power of productivity than species richness. This indicated that functional traits of dominant species of all species as the better predictor of ecosystem properties and processes than species richness^12,35^. In recent work we had reported that fast growing but less competitive forb species dominated communities in early succession, while slow growing but highly competitive species dominated late-successional communities^23^. Moreover, dominant species (forb species) in early succession exhibited relatively high photosynthesis rates and leaf proline content, but showed low seed mass, seed germination rate and SLA, whereas the converse were true for dominant species (graminoid species) in late successional communities^22,23^. In addition, these five traits demonstrated significant trait convergence during succession^19^. Thus, CWMs but not FDs for these five traits may determine productivity during succession. Indeed, our variance partitioning results demonstrated that CWMs for these five traits explained large proportions of productivity for all successional meadows, whereas FDs for these five traits explained much less. Such a pattern has been reported at least once before, albeit for old-growth tropical forest communities. These results indicated that superior performers (dominant traits) but not complementary plant strategies play an important role in determining productivity in these successional meadows. Moreover, it was the biomass of forb and graminoid species that was highest in early and late succession, respectively. This turnover in the dominant plant taxa from early to late succession clearly points to the role of dominant traits that have the major influence on productivity too. As a result, the functional traits of dominant species (CWMs) but not species richness and niche complementarity (FDs) determined productivity in these meadow plant communities.

**Figure 5:**
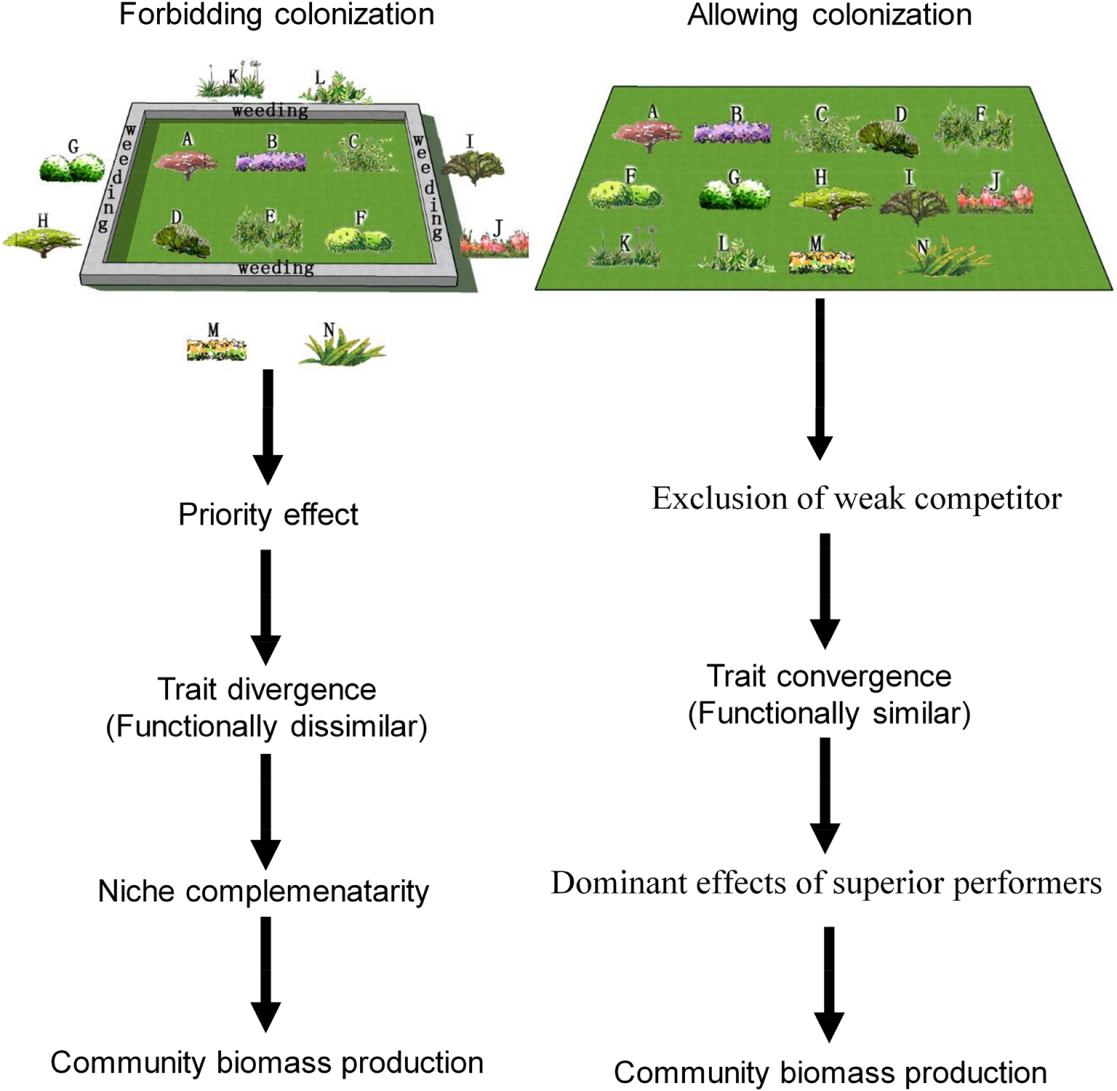
Hypothesized influence of forbiddening and allowing species colonization on the relative importance of niche traits of dominating species in productivity.

Although it is not possible to identify the mechanism driving the diversity–biomass relationships based on the data we have, our results appear contrary to the crucial role of ‘niche complementarity’ effects in productivity over time in experimentally manipulated short-term communities^2,13^. As illustrated in Fig. 5, since new species colonization is disallowed by weeding in experimental communities, the variety and complementarity of species (FD) would tend to determine productivity^17^. Moreover, in most cases, competitive exclusion is prevented^17,36^, so this experimental scenario is less likely to detect the effects of the traits of dominant species on productivity. These may be the reason why ‘niche complementarity’ has often been found to be the key driver of the relationship between plant diversity and productivity through time in experimentally manipulated communities^13^. When species colonization was allowed in experimental communities, trait convergence (functional similarity) appeared to emerge^37^, signaling perhaps the exclusion of weak competitors by the arrival of species with dominant traits^38^. Similar trait convergence has been found in naturally assembled communities where species colonization is always allowed^19,39^. Thus, niche complementarity may determine productivity in the absence of species colonization, whereas traits of dominating species tend to assume greater importance for productivity in the presence of species colonization. Our results therefore indicate that strong positively diversity–productivity relationships in the natural sub-alpine meadow communities across time are up to superior performers in naturally developed communities where colonization occurs. These results, and the similar findings in a tropical forest, suggest that further work is needed to clearly identify the mechanisms that connect diversity to productivity, particular for communities that are influenced by the full range of assembly processes. Moreover, the influences of soil factors on diversity–productivity relations merits future investigation too.

## Conclusion

Overall, our study brings new insights into how species richness, functional trait diversity, and functional traits of dominant species affect productivity in naturally assembled communities across time. On one hand, our results expand the scope of positive diversity–productivity relationships from artificial experiments of short duration in grasslands to natural long-term meadow communities. However, these positive relationships between species diversity and productivity were attributed to the consistent effects of the dominant traits or species in the community.

## Supplementary information

Supplementary Information.
