# Supplementary material for: Strong positively diversity–productivity relationships in the natural sub-alpine meadow communities across time are up to superior performers
Source: Sci Rep. 2020 Aug 7;10:13353. doi: 10.1038/s41598-020-70402-6 (PMC7414895; doi:10.1038/s41598-020-70402-6)
Supplement: Supplementary file 1 — Supplementary Information. [file 41598_2020_70402_MOESM1_ESM.doc]

**Strong positively diversity-productivity relationships in the natural sub-alpine meadow communities across time are up to superior performers**

Kai Jiang^1,2#^, Zhaoyuan Tan^1,2#^, Qifang He^1,2#^, Lu Wang^1,2^, Yang Zhao^1,2^, Xinhang Sun^1,2^, Weichen Hou^1,2^, and Wenxing Long^1,2^, Hui Zhang^1,2*^

^1^College of Forestry / Wuzhishan National Long Term Forest Ecosystem Monitoring Research Station, Hainan University, Haikou 570228, P. R. China

^2^Key Laboratory of Genetics and Germplasm Innovation of Tropical Special Forest Trees and Ornamental Plants (Hainan University), Ministry of Education, College of Forestry, Hainan University, Haikou 570228, P. R. China

**Running Head:** interrelationships among plant functional traits, richness, and productivity

^#^ These authors contributed equally.

**^*^Correspondence author:**

Hui Zhang

Tel: +86-13925183735

mail: 993781@hainu.edu.cn

**Text S1.** Methods for functional trait measurements

***Measurements of plant height, and specific leaf area***

According to the protocols and methods of Cornelissen et al.^1^, we measured plant height (the shortest distance between the upper boundary of the main photosynthetic tissue of the plant and the ground level) of each aboveground ramet for all species in each quadrat. Twenty of the second or third leaves from 15-20 individuals for all species in each quadrat were collected, stored in sealed plastic bags, and immediately transported to the laboratory. Leaf area Leaf size (cm^2^) was determined using a scanner (CanoScan LiDE 700F), and analyzed with an image processing software (ImageJ, version 1.43u, National Institute of Mental Health, Bethesda, Maryland, USA). Leaves were then oven-dried at 80℃ for 2 days and weighed to determine leaf dry mass (W). Leaf specific area (SLA) was calculated as leaf area (cm^2^) per unit of W (g).

## Measurements of photosynthetic rate, and leaf proline content

At each site, the maximum photosynthetic rate of each species in each site was measured between 9:00 am and 12:00 am during sunny days, with a portable photosynthesis system (Li-6400, Li-Cor, Lincoln, Nebraska, USA). Based on preliminary trials^2^, the photosynthetic photon flux density was set at 1500 µmol m^-2^ s^-1^ to ensure that light-saturated photosynthetic rates were measured for all species. Ambient CO_2_ and air temperature were maintained at 370 µmol mol^-1^ and 26°C, respectively. Five leaves from different individuals were selected per species for photosynthetic measurements.

To measure the leaf proline content, we collected 20 leaves for each species. The second or third leaf from as many as 20 individuals was sampled from separate quadrats, if species abundance and frequency of occurrence permitted this. The proline content of leaves was measured as described by Marín et al.^3^, by the following 4 procedures: 1 ) approximately 0.5g of plant material was homogenized in 10 ml of 3% aqueous sulfosalicylic acid and the homogenate filtered through filter paper; 2) Two ml of filtrate was reacted with 2 ml acldninhdrin and 2 ml of glacial acetic acid in a test tube for 1 hour at 100°C, and the reaction terminated in an ice bath; 3) The reaction mixture was extracted with 4 ml toluene, mixed vigorously with a test tube stirrer for 15-20 sec; 4) The chromophore containing toluene was aspirated from the aqueous phase, warmed to room temperature and the absorbance read at 520 nm using toluene for a blank; 5) The proline concentration was determined from a standard curve and calculated on a fresh weight basis as follows: [(µg proline/ml × ml toluene) / 115.5µg/µmole]/[(g sample)/5] =µmoles proline/g of fresh weight material.

***Determination of seed mass and germination rate***

Seeds were collected from meadows that were in close proximity to the sampling sites at the onset of their dispersal period (August to October). We traveled throughout the whole study areas for each successional meadow to collect seeds of as many species as possible, to ensure that our database would represent the entire community. Seeds collected for a particular species were derived from more than 20 individual plants, except when the species was rare. Soon after collection, we cleaned and air-dried the seeds and stored at 20°C. Seed mass was determined by weighting 100 seeds for each species tested in this study. To determine the seed germination rate, we examined the seed viability of each species using the triphenyl tetrazolium chloride test (TTC) prior to seed germination experiments^4^. The seed germination experiments were conducted in incubators (Conviron E15 Growth Chamber, Controlled Environments Ltd., Winnipeg, Canada) at two alternating temperatures [25°C (12h)/5°C (12h), 20°C (12h)/10°C (12h)] and one constant temperature (15°C). At the study site, species either germinate from late March to late May in spring or from late August to late October in autumn. However, the daily temperature range is about 5-25°C near the soil surface in large gaps and about 10-20°C near the soil surface under vegetation in both spring and autumn germination season. Thus, the alternating temperatures 5/25°C and 10/20°C were selected to approximate daily temperature regimes in a large gap and under vegetation, respectively. The constant temperature 15°C was selected as control treatment of the two alternating temperatures. These temperatures regimes have the same average temperature (15°C). Thus, we could determine the differences between germination of seeds tested at an alternating temperature with large amplitude, an alternating temperature with small amplitude and a constant temperature. For each species, there were three replicates of 50 randomly-selected seeds, which were incubated on filter paper moistened with distilled water in Petri dishes (9 cm in diameter) in darkness; with a relative humidity within the chambers of ~70%. The seeds were checked daily for germination, at which time they were exposed to light for several minutes. Thus, any light requirement by the seeds was likely fulfilled during these exposures^5^. Germinated seeds (radicle visible) were removed from the Petri dishes at each counting, and water was added to the filter paper as required. To control for mold infections, when we checked for germination, and also checked for fungi. If any fungal infections were detected on the seeds, we rinsed them with distilled water and subsequently introduced them into a new Petri dish. The duration of the germination test was 60 days.

**References**

1. Cornelissen, J. H. C. et al. A handbook of protocols for standardized and easy measurement of plant functional traits worldwide. *Aust. J. Bot.* **51,** 335-380, http://doi.org/10.1071/bt02124 (2003).
2. Zhang, H. et al. Shifts in functional trait–species abundance relationships over secondary subalpine meadow succession in the Qinghai-Tibetan Plateau. *Oecologia* **188,** 41-47, http://doi.org/10.1007/s00442-018-4230-3 (2018).
3. Marín, J., Andreu, P., Carrasco, A. & Arbeloa, A. Proline content in root tissues and root exudates as a response to salt stress of excised root cultures of Prunus fruit tree rootstocks. *ITEA. Durante* **105,** 282-290 (2009).
4. Ruf, M. & Brunner, I. Vitality of tree fine roots: reevaluation of the tetrazolium test. *Tree. Physiol.* **23,** 257-263, http://doi.org/10.1093/treephys/23.4.257 (2003).
5. Baskin, C. C. & Baskin, J. M. *Seeds: ecology, biogeography, and evolution of dormancy and germination.* Academic press, San Diego. (1998).
